# Disruptive Technologies for Learning and Further Investigation of the Potential Toxicity Produced by Titanium in the Human Body during the COVID-19 Pandemic Period

**DOI:** 10.3390/toxics11060523

**Published:** 2023-06-09

**Authors:** Mădălin Dorel Țap, Cristina Stanciu (Neculau), George Popescu, Octavia-Sorina Honțaru

**Affiliations:** 1 Faculty of Dental Medicine, “Titu Maiorescu” University of Bucharest, 031593 Bucharest, Romania; tapmadalin.dr@gmail.com; 2 Department of Marketing and Medical Technology, University of Medicine and Pharmacy “Carol Davila” Bucharest, 020021 Bucharest, Romania; 3 Emergency Clinical Hospital Dr. Bagdasar-Arseni, Șoseaua Berceni 12, 041915 Bucharest, Romania; 4 Faculty of Sciences, Physical Education and Informatics, University of Pitesti, Târgul din Vale 1, 110040 Arges, Romania; sorina.hontaru@upit.ro; 5 Department of Public Health Arges, Exercitiu 39 bis, 110438 Arges, Romania

**Keywords:** disruptive technologies, oral implantology, pharmaceutical field, titanium toxicity, COVID-19, digital healthcare

## Abstract

Titanium is considered to be a biocompatible material and is used to a great extent in the pharmaceutical and oral implantology fields. While initially, specialists considered that its use does not cause adverse effects on the human body, as time has gone by, it has become clear that its use can lead to the development of certain diseases. The objective of this study was to identify the way in which digital technologies have the capacity to facilitate information regarding the potential long-term harm caused by titanium device toxicity during the COVID-19 pandemic. In this study, a regression model was developed to identify how a series of independent variables have the ability to influence the dependent variable (respondents’ perceptions of how new web technologies have the ability to help future physicians to facilitate information absorption with regard to potential titanium toxicity). The results illustrated that new technologies have the potential to support both the learning process on this topic and the innovation activity by discovering new solutions that will gradually lead to the reduction of the side effects of titanium used in the pharmaceutical and oral implantology fields.

## 1. Introduction

Titanium is considered to be one of the most widely used materials in the medical and pharmaceutical fields both because of its very strong strength over time and its biocompatibility, but also because of the history it has in both fields [1]. The use of titanium in these sectors is mostly due to its very high success rate, and minimal failures but also very good results achieved over the years. Looking at its use in oral implantology, it should be noted that this material began to be used for medical devices in 1952 when Professor Branemark carried out the first research into its compatibility with the human body [2]. Today, six types of titanium are used in this field to make medical devices. Four of them are pure titanium grades (I, II, III and IV), while two of them are made of titanium alloys: Ti-6Al-4V and Ti-6Al-4V-ELI [3].

The use of titanium in oral implantology brings a number of advantages, among which we would like to mention the following: it has a fairly high melting point (1688 degrees), which facilitates its sterilization; its density is low even if its hardness is higher [4]; medical devices made of this material are characterized by high strength; it is non-magnetic and presents ease of handling; medical devices made of this material (in oral implantology) are the best option for patients as they achieve the best results in the osseointegration process; and there is a variety of offers in terms of medical devices made of titanium and their costs are lower compared to those made of other metals.

In addition to these benefits outlined above, there are also various possible disadvantages of implants generated from this material. In the studies that were developed in the past, the authors mentioned that titanium implants can cause some medical problems (even if they were not frequently met). Over time, several studies have been made on both humans and animals [5] to identify the toxicity of titanium. Even if the results illustrated that titanium implants do not present high toxicity for the human body, there were specialists who specified that dental implants may cause some complications. These aspects regarding the possible toxicity of titanium were not accepted by all dentists. Previous studies have illustrated the fact that the possible adverse reactions that may occur due to titanium toxicity (even if some of them are met in rare circumstances) include corrosion over time [6,7], which can lead to instability or loss of the implants [8]; the occurrence of hypersensitivity to titanium, which can lead to a range of allergies [9,10]; and peri-implantitis [11]. From a pharmaceutical perspective, it should be noted that titanium dioxide (TiO2) is regularly used in this field. It first appeared a century ago and is characterized by very good chemical properties, low cost and high availability. It is a naturally occurring oxide of titanium and is considered to have minimal toxicity and low biological effects. Since it was classified as a bio-inert material, it has started to be increasingly used in the pharmaceutical field [12].

### Disruptive Technologies That Can Be Applied in the Future and in the Further Investigation of the Possible Side-Effects of Titanium in the Human Body

Education is the key to success in any field of work, especially dentistry. Through the educational system, young students have the opportunity to acquire the knowledge and skills necessary for the future success of their work. A strong education of students before they have their first contact with patients is very important for doctors, as well as pharmacists because this is the focal point of their entire career [13].

The passage of time as well as the development of new technologies have led to the emergence of new teaching–learning methods and techniques in the online environment. The emergence of online platforms has provided the opportunity for medical and pharmacy students to download a range of educational materials and conduct a series of interactive simulations in the online environment [14]. It is well known that action learning [15] is considered to be the most important in this field because the goal of teaching–learning activity in the two fields is that those who participate in the courses acquire knowledge, skills and abilities that they can put into practice later. For this reason, interactive simulations via e-learning platforms are very important, as they are designed to develop students’ clinical skills [16].

Robots are machines that have the ability to perform certain tasks automatically programmed by a computer. They are increasingly being used in a variety of industries, including oral implantology and pharmaceuticals. It is now recognized that robots are used in various departments in oral implantology, but according to specialists in the field, they are frequently used to carry out the work of nurses, as it has been observed that the work carried out by surgeons cannot be easily replaced [17]. Robots are also used to perform the hygiene part of both medical and pharmaceutical research offices. The passage of time has illustrated that fatigue as well as the pressure that both doctors and pharmacists are under can lead to medical errors. Thus, they have also started to be used to analyze the possibility of combining certain substances, to identify adverse effects that may occur with the use of certain drugs, to test certain drugs/vaccines, to diagnose certain conditions, to make appointments and to perform various surgical operations in the field of implantology [18].

In the last decade, IoT technology has started to be increasingly used in the medical and pharmaceutical industry, with applications developed to facilitate the identification of innovative pharmaceutical solutions, prevent certain diseases and effectively treat certain conditions [19]. Cloud technology, smartphones and new apps in the market have helped both doctors and pharmacists to continuously monitor the diseases that patients suffer from and provide them with the best treatments or the best suggestions in terms of medicines they can take to treat different conditions [20].

IoT technology [21] has started to be increasingly used in the field of oral implantology to be able to reduce the cost and turnaround time of medical procedures [22], to improve communication with patients, but also to provide them with the best information in real time. Some specialists use IoT technology to reduce patient waiting times. They use smart apps that provide them with real-time information about doctors’ availability. In addition, through them, they can contact the doctor directly and make an appointment directly through the app. Moreover, through IoT, doctors are able to check the real-time health status of patients as well as the progress of a particular treatment. This technology also helps to reduce errors in the field, as well as collect data on patients’ conditions, prescribed treatments and their progress over time [23].

The application of artificial intelligence in both oral implantology and pharmaceuticals is very important [24]. AI is intensively used in dental radiology, where it provides detailed information on various conditions the patient suffers from [25]. Another problem that has arisen in the field of oral implantology and is being addressed by AI-based applications concerns the difficulty of identifying the type of medical device that has been used on a patient. Over time, a number of AI models have been developed to identify the type of medical device an individual is using based on periapical and panoramic radiographs [26]. In the field of oral implantology, AI technologies are also used to produce a specialized software-based guide to detect bone thickness, height and density based on chronic beam CT data. This information is essential for clinicians when making the decision to perform medical device placement [27]. AI has also recently been used to be able to analyze the successful osseointegration of a medical device but also to be able to make predictions about its strength over time. At the level of the AI models that have been created, a number of risk factors related to the patient and the conditions they suffer from, as well as a number of other criteria, have been analyzed so that a clear conclusion can be drawn from this data [28].

Virtual reality is an advanced technology that aims to mimic the environment in a three-dimensional replica, making the individual feel as if they are at the center of that environment and have the ability to interact personally with certain tools, based on a series of special technologies. In both the oral implantology and pharmaceutical fields, augmented reality has started to be increasingly practiced, with both doctors and pharmacists using special glasses and an integrated screen where they can visualize all the data they need to perform certain procedures. The main data that can be visualized on such devices are the parameters of the results obtained, the chemical combinations that can be achieved, 3D reconstruction, etc. [29].

Three-dimensional printing (3D printing) technology was first used in 1984 [30] by Charles Hull, and later Hull built the first 3D printing system. Since that time, 3D printing has started to be increasingly used in various fields of activity, as it solves a number of existing problems in various markets [31]. Three-dimensional printing technology has recently been used in many different fields of activity [32], including oral implantology and pharmaceuticals. The role of this technology in both fields is to produce certain individualized materials that meet the existing needs of each individual patient [33].

In the pharmaceutical field, the use of 3D printing technology is very important especially when making new drugs. Thus, when clinical formulation takes place, 3D printing over the solution for rapid prototyping. This automatically leads to the provision of important information on the results obtained and the aspects that could be improved. All these aspects lead, over time, to a reduction of production costs and time to market for new drugs [34].

## 2. Materials and Methods

### Scope of the Research and Survey Design

Given the importance of digital technologies both in the field of dental implantology and in the pharmaceutical field, it was considered necessary to conduct a quantitative study to identify the opinion of future clinicians on how new disruptive technologies can help them to more easily assimilate information on titanium toxicity and adverse effects on the human body in the pandemic era. For this purpose, a regression model was developed to study how the dependent variable (respondents’ perception of how new technologies can help future doctors to assimilate titanium toxicity information more easily) is influenced by the independent variables that were considered in the regression model. The hypotheses underlying the research are as follows:

**H1.**  *Respondents’ perceptions of how new web technologies have the ability to help future physicians assimilate titanium toxicity information more easily are directly and positively influenced by the ease of use of these technologies;*

**H2.**  *Respondents’ perceptions of how new web technologies have the capacity to help future physicians more easily assimilate titanium toxicity information are directly and positively influenced by the accessibility of web technologies;*

**H3.**  *Respondents’ perceptions of how new web technologies have the capacity to help future physicians more easily assimilate titanium toxicity information are directly and positively influenced by the quality of the information provided by these technologies;*

**H4.**  *Respondents’ perceptions of how new web technologies have the capacity to help future physicians more easily assimilate titanium toxicity information are directly and positively influenced by physicians’ responsiveness to use new technologies at the learning process level;*

**H5.**  *Respondents’ perceptions of how new web technologies have the ability to help future physicians more easily assimilate titanium toxicity information are directly and positively influenced by the experience future physicians have in using new digital technologies;*

**H6.**  *Respondents’ perceptions of how new web technologies have the ability to help future physicians more easily assimilate titanium toxicity information are directly and negatively influenced by the cost of purchasing new technologies;*

**H7.**  *Respondents’ perceptions of how new web technologies have the ability to help future physicians easily assimilate titanium toxicity information are directly and negatively influenced by the security risks of web Technologies.*

Data were collected using a questionnaire that was posted between January and March 2022 on an online platform and subsequently distributed to respondents. Respondents were able to access a link and answer the questions in the questionnaire. The sampling method used was snowball sampling. Regarding the way in which the questions were structured, it should be noted that as the basis of the questionnaire, there were a series of questions aimed at building up the demographic profile of the respondents. The first question was a filter question, which had the purpose of selecting only the students who are part of the group under investigation. The following questions were designed to fulfill the purpose of the research. The study was conducted on a sample of 104 students enrolled at the time of the study in the Faculty of Pharmacy and the Faculty of Dental Medicine of the University of Medicine and Pharmacy “Carol Davila”.

Regarding the demographic profile of the respondents, it should be noted in Table 1 that 88.5% of those who participated in the study were women, while 11.5% of them were men. Analyzing in terms of the age distribution of the respondents, most of them (72.1%) are aged between 23–27 years, while 27.9% of them are aged between 28–32 years. If we analyze from the perspective of the faculty in which the students are enrolled, 46.2% of them attend the Faculty of Pharmacy while 53.8% of them attend the Faculty of Dentistry. Studying from the perspective of the respondents’ environment of residence, 90.4% of them live in urban areas, while 9.6% of them live in rural areas.

As for the regression model that was carried out in this study, it was based on the following formula: Formula 1. Y= β0 + β1 × X1 + β2 × X2 + β3 × X3 + β4 × X4 + … + βn × Xn + Ɛ

The indicators present in this formula are as follows: the dependent variable (Y); the independent variable (β); the constant (β0); and the standard error (Ɛ).

Applying the regression formula in the proposed model, the following emerges:

Respondents’ perceptions of how new web technologies have the capability to help future physicians assimilate titanium toxicity information easier = β0 + β1 × The experience future doctors have in using new web technologies + β2 × the ease of use of new web technologies + β3 × accessibility of new web technologies + β4 × quality of the information provided through new web technologies + β5 × responsiveness of doctors in using new web technologies + β6 × security risks of new web technologies + β7 × the cost of new web technologies + Ɛ

## 3. Results

The first objective of this research was to identify the opinion of young people regarding titanium toxicity. Regarding the toxicity of titanium dioxide used in the pharmaceutical field, 65.4% of young people stated that the use of titanium dioxide can have negative effects on the human body, while 34.6% of them consider that it is safe and does not produce negative side effects over time.

Following the interpretation of the results, it was observed that 76.9% of those who participated in the study considered that the use of titanium and titanium alloys in oral implantology can be toxic to the human body, while 23.1% of them disagreed with this aspect, considering that titanium is a material biocompatible with the human body and does not produce negative side effects on individuals.

**Figure 1:**
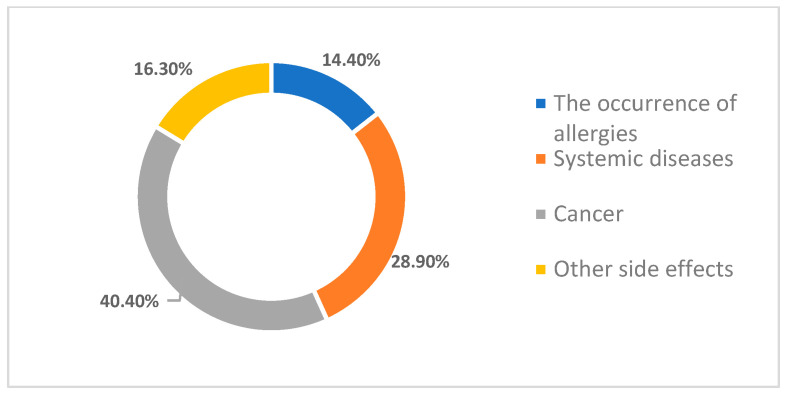
Adverse effects that may arise from the use of titanium dioxide in pharmaceuticals.

Another aspect that was studied in this work was to identify the students’ opinions on the adverse effects that may occur from the use of titanium dioxide in pharmaceuticals. Respondents indicated that in their opinion, the development of various forms of cancer is the main side effect of titanium dioxide toxicity (40.4%). Further, 28.9% of them believe that titanium dioxide can cause systemic diseases over time, while 14.4% of them believe that titanium dioxide can cause certain allergies. Finally, 16.3% of those who participated in the study indicated other adverse effects (Figure 1).

**Figure 2:**
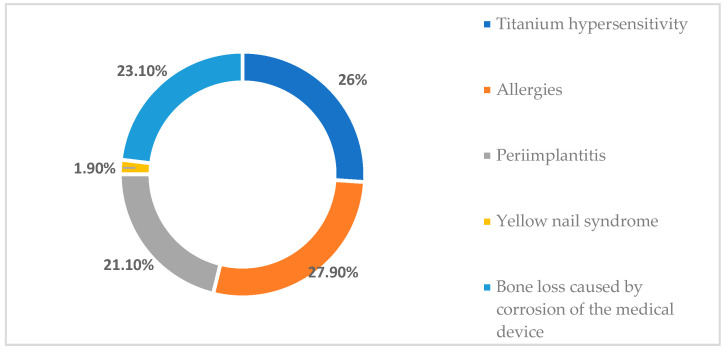
Adverse effects that may occur following the use of titanium in oral implantology.

Regarding the respondents’ perceptions of adverse effects that may occur due to the use of titanium in oral implantology, in Figure 2 it should be noted that 27.9% of the respondents indicated that titanium can cause some allergies; 26% of the respondents indicated that the use of titanium in dental implants can cause titanium hypersensitivity; and 21.1% of the respondents indicated that peri-implantitis is one of the most common adverse effects in oral implantology following a titanium implant. Moreover, 23.1% of those who took part in the study considered that one of the most common adverse effects in practice is bone loss due to corrosion of the titanium dental implant, while only 1.9% of them believed that the use of titanium in oral implantology can cause yellow nail syndrome (Figure 2).

**Figure 3:**
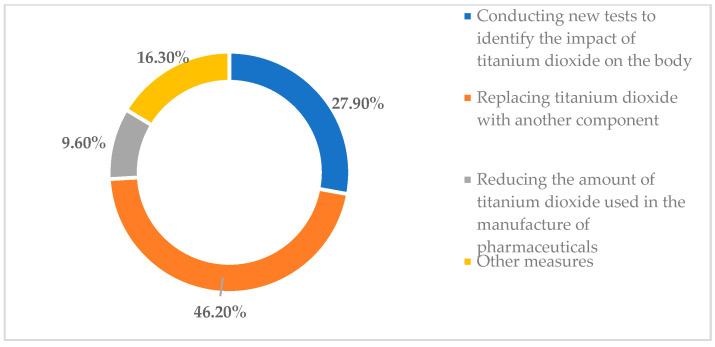
Measures that should be taken to reduce adverse effects that may arise from the use of titanium in pharmaceuticals.

Based on the adverse effects that can occur from the use of titanium in the pharmaceutical field but also in oral implantology, the subsequent aim was to identify the respondents’ perceptions of what measures should be taken in the future to reduce the side effects arising from the use of titanium, titanium dioxide and titanium alloys. The analysis was carried out from two perspectives, namely, from the point of view of adverse effects that may arise from the use of titanium dioxide in the pharmaceutical field and from the point of view of adverse effects that may arise from the use of titanium and titanium alloys in oral implantology. From a pharmaceutical perspective, 46.2% of the respondents indicated that titanium dioxide should be replaced by other components in order to reduce its harmful effects on the human body. In addition, 27.9% of the young students believed that further clinical studies should be carried out to further analyze the impact of titanium dioxide on the human body; 9.6% of them believed that in order to reduce the adverse effects of titanium dioxide on the human body, the amount of titanium dioxide currently used in pharmaceuticals should be reduced; and 16.3% of those who took part in the survey indicated other measures that should be taken (Figure 3).

**Figure 4:**
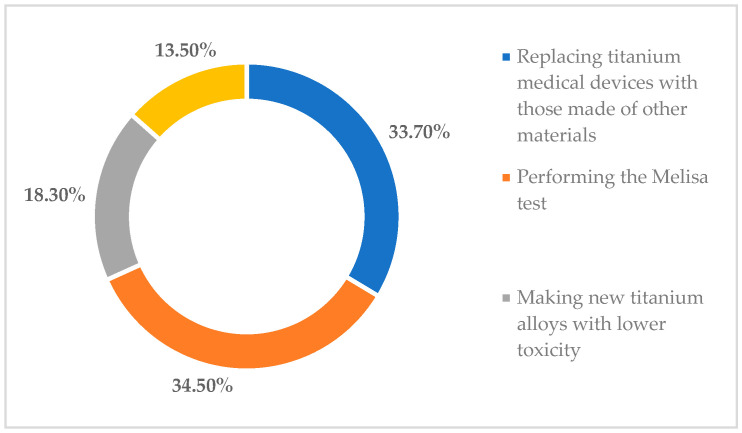
Measures that should be taken to reduce adverse effects that may arise from the use of titanium in oral implantology.

In terms of the measures that should be taken in the field of oral implantology to reduce the negative impact of titanium on the human body, in Figure 4 it should be noted that 34.5% of the participants in the study believe that Melisa tests should be carried out to identify the sensitivity of patients to metals; 33.7% of them stated that in individuals who show adverse reactions, dental implants made of titanium should be replaced by those made of ceramic (e.g., zirconium), which have fewer adverse effects; while 18.3% said that the development of new titanium alloys with lower toxicity is the most effective measure to reduce the adverse effects that may occur later. Finally, 13.5% of respondents mentioned other measures that should be taken in the field of oral implantology to reduce the impact of titanium on the human body.

**Figure 5:**
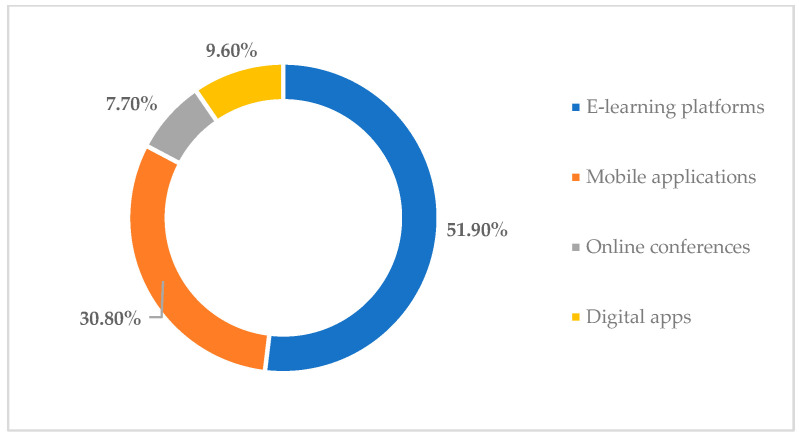
Digital technologies used by respondents so far to learn about titanium toxicity.

Moreover 89.4% of those who took part in the survey believed that there is a need for further study in the faculty on the issues of titanium toxicity and the adverse effects that may arise from this problem. In addition, they also consider it necessary to study in depth the results concerning the measures that can be taken and the treatments that should be followed to reduce the negative effects of this metal on the human body. Another aspect that has been studied concerns the technologies that the young students have used so far to learn about the toxicity of titanium and how it can produce side effects on the human body. Further, 51.9% of those who took part in the study indicated that they had researched the topic from e-learning platforms that provided them with a range of materials in digital format and were thus able to learn more about the topic; 30.8% of them mentioned that they used mobile apps; 9.6% of them used digital apps; and 7.7% of them attended conferences held online, as a result of which they obtained more information on the topic (Figure 5).

**Figure 6:**
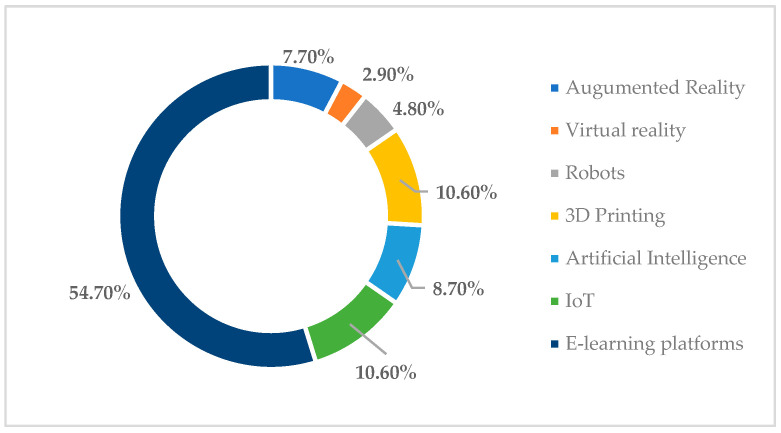
Respondents’ perceptions of technologies that can be used to facilitate further information on titanium toxicity.

Regarding respondents’ perceptions of the technologies that should be used to facilitate further information on titanium toxicity and the adverse reactions that may occur as a result, in Figure 6 it should be noted that 54.7% of students considered e-learning platforms to be the most effective in this regard, while 10.6% of them believed that applications based on IoT or 3D printing technologies are very helpful in research–learning, especially when it comes to identifying how to reduce adverse effects caused by the use of titanium in the production of dental implants. Further, 8.7% of respondents felt that artificial intelligence can make it easier to assimilate new information in the field, 7.7% felt that augmented reality technology is the most effective, and 4.8% felt that robots can help specialists to learn more about the subject. Only 2.9% of those who took part in the survey felt that virtual reality could make it easier to gain insight into titanium toxicity.

**Figure 7:**
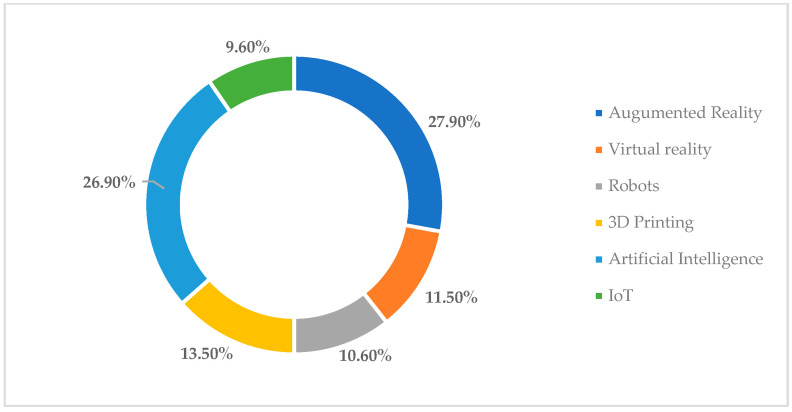
Respondents’ perceptions of technologies that can be used to develop new innovative solutions to reduce the negative effects of titanium use on the human body.

In terms of respondents’ views on technologies that can be used to identify new solutions to reduce titanium toxicity, 27.9% of respondents felt that augmented reality technology could be of great help in determining new medical devices or identifying treatments to reduce the harmful impact of titanium on the human body; 26.9% of them thought that artificial intelligence is the best option in this respect; 13.5% opted for 3D printing; while 11.5% thought that virtual reality can help specialists to find a solution to the problem more easily. Finally, 10.6% of respondents mentioned that robots can be involved in research to determine new solutions to reduce titanium toxicity, while 9.6% of respondents opted for IoT-based applications (Figure 7).

Regarding the main indicators that were obtained at the level of the regression model, in Table 2 it should be noted that the value of the R indicator was 0.717, while the value of R Square was 0.514. This suggests that 51.4% of the variation of the dependent variable is explained by the independent variables. The value of the standard error was 1.112. In terms of degrees of freedom, the value of df1 is 7 while that of df2 is 96. The value of F Change is 14.481. Since the value of Sig. is less than 0.05, the proposed model is considered to be valid.

Table 3 shows the values that were obtained at the coefficient level. For the regression model that has been proposed, only coefficients whose Sig. value is less than 0.05 will be considered. From this point of view, the coefficients that will be included in the analysis are the experience that future doctors have in using new web technologies; the ease of use of new web technologies; the quality of the information provided by new web technologies; the responsiveness of future doctors in using new digital technologies; and the security risks perceived by the respondents and the cost of new technologies. It can be seen that for the coefficient of accessibility of web technologies, the value of Sig. is 0.94 > 0.05 and as a result, this coefficient will not be taken into account in the regression model.

Applying the values obtained from the analysis carried out on the regression model level, we obtain the following.

Respondents’ perceptions of how new web technologies have the potential to help future physicians assimilate titanium toxicity information more easily = 4.877 + 0.582 × the experience future doctors have in using new web technologies + 0.297 × ease of use of new web technologies + 0.235 × quality of the information provided through new web technologies + 0.538 × responsiveness of doctors in using new web technologies − 0.272 × responsiveness of future doctors to use new web technologies − 0.528 × cost of new web technologies + 1.112

## 4. Discussion

Titanium dioxide has been used for many years in the manufacture of pharmaceuticals. Expert opinion is divided on the side effects that can occur from its use. A similar situation is found in the field of oral implantology, where titanium and titanium alloys are used to make dental implants. Studies conducted over the years have illustrated that, over time, the use of titanium can lead to the development of titanium hypersensitivity [35] allergies or systemic diseases. Based on these aspects, in recent years there has been a growing desire among specifiers to assimilate information on this issue.

Since its use in the medical and pharmaceutical fields, titanium has been considered to be a biocompatible material in the human body [36]. Theoretically, a material is considered to be biocompatible when metal ions (which are dissolved from metals in the human body) do not have the ability to react with human cells or biomolecules, producing certain changes in their functions. Under these conditions, metal ions are considered to be non-toxic to the human body. Recent studies in this field have shown that none of the materials currently used in medical devices are fully biocompatible; in other words, they do not affect the human body at all [35]. Over time, it has been observed that titanium implants can cause certain problems over time (even if they were identified in a limited proportion, just a few cases registered).

Identifying the problem of titanium release into the body, several specialists in the field have tried to identify new ways in which they can reduce or eliminate the release of titanium into the human body. However, regardless of the techniques used to prevent this, they failed to achieve any concrete results. Even though titanium is considered to be one of the most important materials in the medical and pharmaceutical field, the technique is constantly concerned with optimizing its biocompatibility and mechanical properties. The emergence of the online environment as well as the concern of specialists in discovering new technologies to support pharmaceutical and implantology research innovation has led to the rapid development of new innovations that support individuals in assimilating information on titanium toxicity more easily. These emerging technologies in the above-mentioned fields have supported both the learning process and have also provided a number of advantages in facilitating processes in these fields, particularly identifying new treatments and new medical devices, facilitating the drug management process, clinical trials, etc. All these aspects have gradually led to cost reductions and increased labor productivity.

On the e-learning platforms, the doctors and the students can view a series of videos to learn how to work with certain medical instruments with new technologies or how to identify new solutions to treat the toxicity of different substances. The COVID-19 pandemic has provided students the opportunity to access these platforms and learn through them how to perform various medical procedures or how to combine certain substances in the making of certain medicines or vaccines. Comparing the information delivered through conventional teaching as opposed to e-learning platforms, it was observed that there is not much difference in terms of the amount of information delivered to learners [37]. In addition, it was observed that they value the courses delivered on online platforms equally compared to conventional ones [38]. A number of technologies can now be used to support the e-learning process. These include computer-based training [39], web-based training, video teaching, etc. Computer-based training refers to all multimedia learning programs that include hypermedia learning materials.

Robots [40,41,42] are not only used to performing easy tasks, but they, along with other technology (e.g., 3D navigation and 3D printing) [43], have the ability to perform much more complex procedures such as invasive dental procedures, which are meant to prepare patients’ teeth and place medical devices with much higher precision. If we are to look at it from a surgical perspective, an important role in this field is played by the experience of the physician as well as the assistance of tomographic imaging [44]. Prakash [45] believes that robots play a vital role in the field of oral implantology as they are meant to increase the accuracy, quality and safety of surgical procedures.

IoT technology is used in dentistry and the pharmaceutical field [46] to the monitoring of the jaw after placement of a medical device can only be done via X-rays. Specialists in this field have developed a sensor made of titanium and polyether ether ketone that can be incorporated into the dental implant and has the ability to measure bone growth permanently, all with the aim of avoiding X-rays. The recorded results are transmitted to a readout device for measurement and interpretation [47]. Artificial intelligence-based technologies are used in this field to discover, design and mass-produce drugs for various conditions and to identify new solutions to treat the toxicity of different substances [48]. In addition, they facilitate the conduct of clinical trials and the selection of the best patients for these clinical trials. Artificial intelligence is intended to improve the quality of the production process and facilitate this process. In addition, it aims to analyze existing data in databases and provide relevant information on the treatments that can be offered to patients to treat various conditions. This technology is also of great importance for determining the treatment needed for rare diseases. In addition, AI facilitates the retrieval and processing of all existing clinical trial data, which facilitates the rapid delivery of results [49].

Recent studies conducted in this field have illustrated that 3D printing helps to improve the fit of biomaterials and medical devices in the human body. The technology has the ability to replicate the complexity of human architecture to a large extent, which causes the materials made to be to a greater extent compatible with each individual’s body [50]. Moreover, it has been observed that 3D-printed dental implants have the ability to bring a number of benefits in terms of osteogenic differentiation as well as early osseointegration after the implantation process has taken place [51]. In terms of placing titanium devices in the human body, recent 3D printing studies have illustrated that, 3D printed material exhibits very good biocompatible performance and because of this it can be used in dental implants [52].

This work aimed to determine the role that new technologies have in facilitating further information on the adverse effects that can occur from the use of titanium in pharmaceuticals and oral implantology in the pandemic era. In order to be able to meet the set objectives, a quantitative study was conducted on a sample of 104 respondents. The analysis of the data showed that the respondents believed that the use of titanium dioxide in the pharmaceutical field and the use of titanium and titanium alloys in oral implantology may cause adverse effects on the human body over time. They believed that the use of titanium dioxide in pharmaceuticals may lead over time to the development of cancers or systemic diseases, while the use of titanium and titanium alloys in oral implantology may lead to the development of titanium hypersensitivity, allergies or periimplantitis. They considered that new technologies can help facilitate learning on this topic and may also be of great importance in the development of new solutions aimed at reducing adverse effects arising from the use of titanium in pharmaceuticals and oral implantology.

Regarding the regression model proposed in this paper, the analysis showed that the respondents’ perceptions of how new web technologies can help future physicians assimilate titanium toxicity information more easily are directly and positively influenced by the experience future physicians have in using new web technologies, the ease of use of these technologies, the quality of information obtained from the use of these technologies, the responsiveness of future physicians to the use of new technologies, and the negatively perceived safety risks of future physicians and the costs of new web technologies.

## 5. Conclusions

Disruptive technologies started to be used in both oral implantology and pharmaceuticals to support drug discovery, reduce human error and facilitate the work of various medical procedures. The two sectors mentioned above are very technology-driven, which has meant that the process of digitalization, specifically to mobile applications and new technologies, has had a strong impact on the doctor–patient relationship. In the field of oral implantology, these technologies have made it possible to assess patients’ problems in detail, their lifestyles and to make correlations that illustrate the main causes that have the capacity to determine the occurrence of certain diseases or complications. This study provides a number of valuable insights for both managers of dental and pharmacy universities and managers who coordinate the activity within specialized clinics, illustrating how new technologies can be used in these institutions to facilitate the uptake of information in various areas of interest, such as possible titanium toxicity, the use of new materials in oral implantology, the toxicology of some pharmaceutical substances.

## Figures and Tables

**Table 1:** Respondents’ profile.

Category		Frequency (%)
Gender	Male	12 (11.5)
	Female	92 (88.5)
Age	23–27 years	75 (72.1)
	28–32 years	29 (27.9)
Faculty where the students are enrolled	Faculty of Pharmacy	48 (46.2)
	Faculty of Dentistry	56 (53.8)
Residence environment	City	94 (90.4)
	Countryside	10 (9.6)

**Table 2:** Main regression model indicators.

Indicators	Validation Criteria
R	0.717
R Square	0.514
Adjusted R Square	0.478
Std. Error of the Estimate	1.112
R Square Change	0.514
Sig. F Change	0.000
df1	7
df2	96
F	14.481

**Table 3:** Coefficients table.

Model	Unstandardized Coefficients		Standardized Coefficients	t	Sig.	Correlations			Collinearity Statistics	
	B	Std. Error	Beta			Zero-Order	Partial	Part	Tolerance	VIF
(Constant)	4.877	0.913		5.339	0.000					
The experience that future doctors have in using new technologies.	0.668	0.168	0.582	3.987	0.000	0.222	0.377	0.284	0.238	4.201
Ease of use of new web technologies.	0.308	0.144	0.297	2.133	0.035	0.273	0.213	0.152	0.261	3.834
Web technologies accessibility.	−0.282	0.167	−0.178	−1.689	0.094	0.300	−0.170	−0.120	0.454	2.201
Quality of the information provided by new web technologies.	0.315	0.144	0.235	2.194	0.031	0.430	0.219	0.156	0.440	2.273
Responsiveness of future doctors to use new web technologies.	0.522	0.103	0.538	5.067	0.000	0.611	0.459	0.361	0.449	2.226
Security risks.	−0.322	0.130	−0.272	−2.469	0.015	−0.013	−0.244	−0.176	0.417	2.397
Cost of new web technologies.	−0.613	0.193	−0.528	−3.183	0.002	0.146	−0.309	−0.227	0.184	5.430

## Data Availability

Not applicable.
